# Prediabetic risk factors among adolescents with obesity

**DOI:** 10.1186/1687-9856-2013-S1-P91

**Published:** 2013-10-03

**Authors:** Satriono, Ratna Dewi Artati, Dasril Daud, Ufy Trisnawati

**Affiliations:** 1 Department of Child Health, Medical School, University of Hasanuddin, Makassar, Indonesia

**Keywords:** adolescents, obesity, prediabetic, blood glucose

## Background

Childhood obesity has increased as a health problem worldwide and WHO have declared that obesity is a global epidemic problem which is needed to be managed promptly. In the last decades, prevalence of childhood obesity has raised dramatically. Childhood obesity may results in overweight and impose other health problems. It is also a risk factor for development of Type-2 Diabetes Mellitus (DM).

## Objective

To determine prediabetic risk factors among adolescents with obesity.

## Methods

The study was conducted as an observational study using cross sectional sectional and case control approaches. Data was collected from level I to III students of two selected Junior High Schools (SMP Katolik Rajawali and SMP Islam Athirah) in Makassar, Indonesia, started on August to October 2011. These students were adolescents with obesity and permissions to participate in the study were gained from their parents. Analysis were done using X2 test or Fisher Exact Test (significat value p ≤ 0,05) as well as logistic regression analysis.

## Results

Data consisted of 114 sample, 75 (65,8%) boys and 39 (34,2%) girls. There were 35 (76,1%) girls and 11 (23,9%) boys with prediabetic. Logistic regression analysis showed that there were 3 factors asssociated with the occurence of prediabetic among adolescents with obesity, which were degree of obesity AOR 4,23 (95% CI 1,92 to 10,77), history of breastfeeding AOR 3,9 (95% CI 1,55 to 9,86) and history of DM in the family AOR 6,48 (95% CI 2,52 to 16,64).

## Conclusion

Degree of obesity, history of breast feeding and history of DM in the family are correlated with prediabetic occurence among adolescents with obesity.

